# Effect of peppermint water on prevention of nipple cracks in lactating primiparous women: a randomized controlled trial

**DOI:** 10.1186/1746-4358-2-7

**Published:** 2007-04-19

**Authors:** Manizheh Sayyah Melli, Mohammad Reza Rashidi, Abbas Delazar, Elaheh Madarek, Mohammad Hassan Kargar Maher, Alieh Ghasemzadeh, Kamran Sadaghat, Zohreh Tahmasebi

**Affiliations:** 1 Department of Obstetrics & Gynecology, Alzahra Teaching Hospital, South Artesh Avenue, Tabriz University of Medical Sciences, Tabriz, Iran; 2 Drug Applied Research Center, University Street, Tabriz University of Medical Sciences, Tabriz, Iran; 3 Department of Pharmacognosy, Faculty of Pharmacy, University Street, Tabriz University of Medical Sciences, Tabriz, Iran; 4 Department of Community Medicine, Faculty of Medicine, Gholgasht Street, Tabriz University of Medical Sciences, Tabriz, Iran; 5 Faculty of Literature and Human Sciences, Azarbayegan University, Tabriz, Iran; 6 Taleghani Teaching Hospital, Rahahan Street, Tabriz University of Medical Sciences, Tabriz, Iran

## Abstract

**Background:**

Nipple pain and damage in breastfeeding mothers are common causes of premature breastfeeding cessation. Peppermint water is popularly used for the prevention of nipple cracks in the North West of Iran. The aim of this study was to determine the effectiveness of peppermint water in the prevention of nipple cracks during breastfeeding in comparison with the application of expressed breast milk (EBM).

**Methods:**

One hundred and ninety-six primiparous breastfeeding women who gave birth between February and May 2005 in a teaching hospital in Tabriz, Iran, were randomized to receive either peppermint water or EBM. Each woman was followed for up to three visits or telephone calls within 14 days and then by telephone call at week six postpartum.

**Results:**

Women who were randomized to receive peppermint water were less likely to experience nipple and areola cracks (9%) compared to women using EBM (27%; p < 0.01). Women who used the peppermint water on a daily basis were less likely to have a cracked nipple than women who did not use peppermint water (relative risk 3.6, 95%CI: 2.9, 4.3). Nipple pain in the peppermint water group was lower than the expressed breast milk group (OR 5.6, 95% CI: 2.2, 14.6; p < 0.005).

**Conclusion:**

This study suggests that peppermint water is effective in the prevention of nipple pain and damage. Further studies are needed to assess the usefulness of peppermint water in conjunction with correct breastfeeding techniques.

Trial registration number: NCT00456404

## Background

Sore nipples are a common complaint among breastfeeding women and one reason why some women decide to stop breastfeeding. The incidence ranges from 11 to 96% [1-3]. Preparation for breastfeeding happens naturally in pregnancy [4], and the presence of 'epidermal growth factor' in breast milk has potential therapeutic benefits by promoting the growth and repair of skin cells [5,6]. Nipple damage may occur due to trauma to the nipple from incorrect attachment to the breast [7,8] and healing may be difficult because of repeated trauma from the infant's suckling [9]. Therefore, the prevention of nipple pain and cracks is important.

A number of reviews have examined the effect of various protocols on either the prevention or treatment of nipple pain and/or trauma [10-12]. Most of the reviewed studies had an inadequate sample size or other methodological problems, which limits the applicability of their findings. Furthermore, the superiority of the creams, sprays, lotions or ointments used for the prevention of nipple crack in lactating primiparous women is in doubt or a scientific basis for their use has not been well-documented [10]. Renfrew and colleagues have stated "There are no grounds for using topical agents to prevent nipple pain and... there are no adequate trials to date" [12] (see p. 29). Some authors have concluded that the use of ointments "may be unnecessary and costly" [13] (see p. 92). However, recently, Tanchev *et al *have shown the effectiveness of purified lanolin in the prophylaxis and treatment of sore nipples [14].

Unfortunately, many women delay seeking treatment until substantial damage already has occurred. Sore nipples heal rapidly, often within a day or two. However, it is still easier to prevent rather than to treat them [15].

Natural remedies in conjunction with medical care may provide relief from breastfeeding problems [16,17]. Peppermint (*Mentha × piperita*) and its oil are used extensively in foods and drugs [18]. Menthol, which is found in the highest concentration in peppermint oil, is pharmacologically active in relatively small doses. In small doses, it is safe for ingestion by babies and has been widely used over many years as a calming agent to soothe an upset stomach. As is observed with numerous other volatile oils, peppermint water possesses antibacterial activity [19]. Because it has calming and numbing effects, it has been used externally for skin anaesthetic, burns, wounds, itching and inflammation. Peppermint water is popularly used for the prevention of nipple pain and damage in Azarbayejan Province, North West of Iran; however, this has not been evaluated previously.

In a pilot study, which we carried out in Alzahra Research and Development Center of Clinical Studies, the topical use of household peppermint water by new breastfeeding mothers was more effective than expressed breast milk in the prevention of nipple cracks (10% compared to 40%). Therefore, we conducted a randomized controlled trial (RCT) to evaluate the effectiveness of a topical preparation of peppermint water in comparison with that of expressed breast milk for the prevention of nipple cracks in primiparous breastfeeding women.

## Methods

### Study design

We recruited women who gave birth in Talegani teaching hospital in Tabriz, East Azarbayejan Province, North West of Iran, where a total of approximately 7,200 women give birth each year. All mothers were given a standardized breastfeeding education with face-to-face demonstrations before starting breastfeeding.

Entry criteria were primiparous women who had given birth to a healthy term infant. Mothers who were discharged before an interview or who had preterm birth, postpartum fever, breast infection, nipple abnormalities, age less than 18 years, twins, taking medications at night, and also who did not have a telephone line or were illiterate, were excluded. Also, the infants who were fed infant formula or used a pacifier, or who had mouth infection or an abnormally short frenulum were excluded. Equal numbers of women who had given birth vaginally and by caesarean section (CS) were recruited.

After giving informed consent, a random numbers table was used to allocate participants (stratified by method of birth) to the experimental group, application of peppermint water after each feed, or the control group, application of expressed breast milk after each feed. The peppermint water group was instructed to put soaked cotton with peppermint water on the nipples and areola after washing the nipples with water following every breastfeed from day 1 to day 14 and wash before the next feed. The same instruction was given to the expressed breast milk group with the difference being that milk was used to soak nipples in place of peppermint water. A single supply of quantified peppermint water was given to all participants of the peppermint water group and they were asked not to use their own household peppermint water or any other medication. Preparation of the peppermint water is described in the Additional file (Preparation of the peppermint water) (See Additional file 1).

### Data collection

Demographic and peripartum information were abstracted from the medical records. An interview was conducted during the postpartum stay.

The follow-up telephone interviews were conducted by a trained midwife at days 4, 8 and 14 postpartum. In the case of nipple or areola crack and pain, both examination of the breast and the scoring were carried out by one researcher according to published methods [10,20]. All mothers were asked about the frequency and duration of breastfeeding at 24 hours and the data were recorded. A follow-up visit was arranged for both groups one week after recruitment (day eight) or at any time during the trial course in the case of nipple crack or pain.

A telephone interview was conducted with all mothers at week six. A questionnaire was used to determine the presence and severity of nipple damage and pain. Each mother scored her own pain during breastfeeding. Rating scales were used to determine the level of pain as follows: no pain, mild (discomforting), moderate (distressing), and severe (excruciating) [10].

The main outcome measures include responses to questions about nipple pain at week six and objective findings from the physical examination at each visit. The physical examination included cracks within and beyond the areola and was expressed in millimeters using criteria used by Amir et al [20]. The nipple damage was defined on the basis of the width of the damage as follows: 1–2 mm, mild; 3–9 mm, moderate; >10 mm, severe and/or a visible yellow color in the crack. Areola damage was also assessed according to the same criteria.

### Sample size

The sample size of the study was determined according to the pilot study by Cochran formula (estimation of percent and ratio) of n=t2pqd2
 MathType@MTEF@5@5@+=feaafiart1ev1aaatCvAUfKttLearuWrP9MDH5MBPbIqV92AaeXatLxBI9gBaebbnrfifHhDYfgasaacH8akY=wiFfYdH8Gipec8Eeeu0xXdbba9frFj0=OqFfea0dXdd9vqai=hGuQ8kuc9pgc9s8qqaq=dirpe0xb9q8qiLsFr0=vr0=vr0dc8meaabaqaciaacaGaaeqabaqabeGadaaakeaacqWGUbGBcqGH9aqpdaWcaaqaaiabdsha0naaCaaaleqabaGaeGOmaidaaOGaemiCaaNaemyCaehabaGaemizaq2aaWbaaSqabeaacqaIYaGmaaaaaaaa@3705@ with a confidence of 95%, where t is the confidence level, p is the ratio of the number of subjects showed nipple crack (n = 18) to the total number of subjects in the pilot study (n = 30), q is the ratio of the subjects did not show nipple crack (n = 12) to the total number of subjects in the pilot study (n = 30), and d is 8% of the obtained value for p [21]. Therefore, the number of subjects in the present study was calculated as: (1.96)^2 ^× 0.6 × 0.4/(0.075)^2 ^= 196.

The measured values were given as mean and standard deviation (SD). Comparison of the categorical variables was made using chi-square tests (χ^2^) when appropriate. Intragroup comparison was carried out using ANOVA test and t-test was used to compare means. Test-retest reliability was used for confirmation of the validity of recorded pain. Odds ratios (OR) and 95% confidence intervals (CIs) were calculated for the independent variables. For all statistical analyses, the differences were considered statistically significant at p < 0.05. The statistical analysis was performed using SPSS program (SPSS 13.0; SPSS, version for windows).

### Ethics approval

The Medical Ethics Committee of Tabriz University of Medical Sciences approved the research study, all participants were given adequate information, and consent was obtained from each participant.

## Results

**Figure 1:**
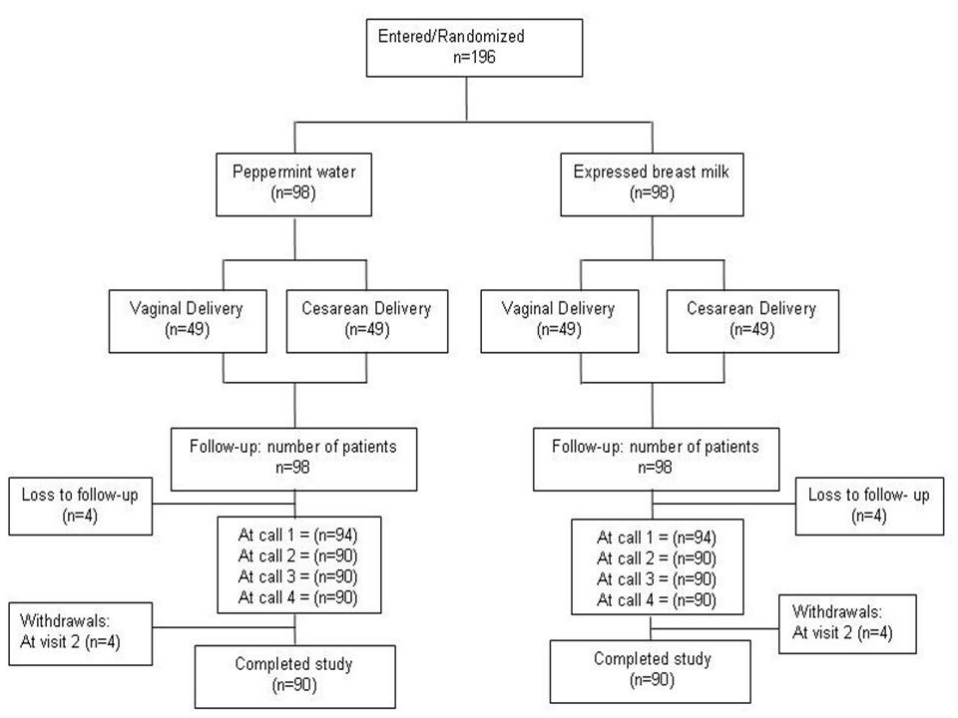
Flow of the study participants.

One hundred and ninety-six mothers who gave birth between February and May 2005 were recruited for the study. We lost eight mothers to the study before the first telephone call. Also, eight mothers were excluded during the course of the study due to infant illness. The CONSORT flow chart is shown in Figure 1.

No differences were found between the two groups (peppermint group and EBM group, respectively) in terms of sex (male; 49% vs. 62%, p = 0.7), and the weight of the infants (3.3 ± 0.4 vs. 3.2 ± 0.4, p = 0.8) by t-test. The demographic characteristics of the participants were also similar in the two groups. At 24 hours postpartum, there were significant differences between peppermint water and expressed breast milk groups with regard to the mean frequency of breastfeeding at 24 hours (10.1 ± 0.9 vs. 10.9 ± 2.1; p < 0.015), and mean duration of breastfeeding at each feed at 24 hours (19.1 ± 5.0 vs. 12.9 ± 3.2 minutes; p < 0.001).

The overall nipple crack rate in the peppermint water and expressed breast milk groups were 7% and 23%, respectively which was statistically significant (p < 0.01) (Table 1). No women in the peppermint water group experienced severe nipple or areola cracks, compared to 13 women (15%) in the breast milk group (Table 1). In the peppermint water group, the nipple crack took place on median of day 7 (6.2 ± 1.9), whereas, in the breast milk group, this was observed on day 4 (3.8 ± 0.9). Only 8 and 19 mothers had a mild or moderate crack, which occurred with delay in the peppermint and EBM groups, respectively. Damaged nipples were less likely in mothers who used peppermint water compared with those who did not (relative risk 3.6, 95%CI: 2.9, 4.3).

**Table 1:** Comparison of the severity of nipple and areola cracks in mothers using peppermint water and expressed breast milk

	**Peppermint water group** **(n = 90)** **n (%)**	**Breast milk group** **(n = 90)** **n (%)**	**p value from χ^**2 **^test**
**Areola crack**			
No crack	88 (98)	85 (94)	0.07
Mild	1 (1)	0 (0.0)	
Moderate	1 (1)	0 (0.0)	
Severe	0 (0.0)	5 (6)	

**Nipple crack**			
No crack	84 (93)	71 (79)	1.01
Mild	3 (3)	3 (3)	
Moderate	3 (3)	8 (9)	
Severe	0 (0.0)	8 (9)	

Overall nipple and areola crack	8 (9)	24 (28)	< 0.001

Nipple pain reported by mothers during the first two weeks of the study has been tabulated in Table 2. Women in the peppermint water group were less likely to report pain (n = 6) than women in the breast milk group (n = 26) (p < 0.001). The breast milk group had a higher odds of experiencing nipple pain than the breast milk group (OR 5.6, 95% CI: 2.2, 14.6).

**Table 2:** Reports of nipple pain in women who received peppermint water and expressed breast milk

**Pain**	**Peppermint water** **(n = 90)** **n (%)**	**Breast milk** **(n = 90)** **n (%)**	**p value from χ^**2 **^test**
0 (No pain)	84 (93)	64 (71)	< 0.001
1 (Mild)	2 (2)	2 (2)	
2 (Moderate)	2 (2)	3 (3)	
3 (Severe)	2 (2)	21 (23)	

During the study, mothers who used peppermint water had a decreased odds of 3.2 (95% CI: 3.02, 6.31) of having pain, while mothers using expressed breast milk had an odds of 0.53 of pain reduction (95% CI: 0.41, 0.68). There were no severe nipple cracks in the peppermint group and the mean ranking for nipple crack was low, but for the expressed breast milk group mean ranking for nipple crack was high (20%) (p < 0.001).

The duration and the frequency of breastfeeding at 24 hours were also assessed according to the type of birth. In the peppermint group, the mean frequency of breastfeeding at 24 hours for vaginal birth and CS were 10.12 and 10.22, respectively. For breast milk groups, the mean duration for each group was 8.72 and 8.08 minutes, respectively. The frequency of breastfeeding in the peppermint group was higher than the EBM group (t-test, p < 0.001). The two-way ANOVA test revealed a significant difference between peppermint water and breast milk groups with regard to the mean duration of breastfeeding for vaginal birth and CS at 24 hours (19.1 vs. 19.24 and 13.8 vs. 11.9 minutes, respectively, p < 0.015). The mean duration of breastfeeding at 24 hours for the peppermint group was higher than the EBM group and was not affected by the method of birth. In the peppermint group, there was no significant difference in the rate of nipple crack between mothers who gave birth by CS or vaginally (p = 0.13). Whereas, the rate of areola crack in mothers who gave birth by CS was significantly lower than mothers who birthed vaginally (p = 0.04).

At week six, in the peppermint group, all participants with vaginal births continued to breastfeed, whereas 13% (n = 6) of the mothers with cesarean delivery used infant formula in addition to expressed breast milk. The corresponding values in the expressed breast milk group were different. In the EBM group, 15% (n = 14) of the cesarean group, and 6% (n = 6) of vaginal birth group used formula in addition to breast milk (χ^2 ^= 3.57, p > 0.05) (Table 3). Six mothers in the expressed breast milk group ceased breastfeeding because of the severity of the nipple crack.

**Table 3:** Outcomes for women using peppermint water and expressed breast milk: objective evidence of nipple and areola cracks, reported pain in the first two weeks, and breastfeeding at six weeks postpartum

**Clinical outcome**	**Peppermint water** **(n = 90)**	**Breast milk** **(n = 90)**	**Odds Ratio**	**95%CI**
Intact nipples and areola	82	66	3.72	1.57, 8.83
Painless feeding	84	64	5.68	2.21, 14.63
Full breastfeeding	84	64	5.68	2.21, 14.63
Partial breastfeeding	6	20	0.25	0.095, 0.65

## Discussion

With adequate support and good information on preventing some of the common problems associated with breastfeeding, a woman's chance of successfully breastfeeding her new baby is greatly improved. There are a number of clinical studies with different treatments and treatment combinations to prevent nipple pain and damage in breastfeeding women [10]. "No one topical agent showed superior results in the relief of nipple discomfort. The most important factor in decreasing the incidence of nipple pain is the provision of education in relation to proper breastfeeding technique and latch-on as well as anticipatory guidance regarding the high incidence of early postpartum nipple pain." [11] (see p. 435). However, no study had examined peppermint water, a common household remedy in Iran, which was thought to prevent nipple pain and damage.

This study showed a significant reduction in the frequency of nipple pain and cracks in breastfeeding mothers where peppermint water was applied after breastfeeds. These effects could be attributed to the calming and numbing effects and the antibacterial activity of peppermint water leading to the reduction of irritation and nipple discomfort. In addition, painful feeding could be the reason for the reduction of frequency and duration of each feed in the expressed breast milk group.

The use of herbs is a time-honored approach to strengthening the body and treating disease [22]. Herbs, however, contain active substances that can trigger side-effects and interact with other herbs, supplements, or medications [22]. For these reasons, herbs should be taken with care, under the supervision of a practitioner knowledgeable in the field of botanical medicine. Pure menthol is poisonous and should never be taken internally. It is important not to confuse oil and tincture preparations. It should be kept away from the eyes and other mucus membranes and should not be inhaled by or applied to the face of an infant or small child [23,24].

As the study was not blinded, a bias could have occurred and influenced the study results. Feeding can be affected by the psychological state of the mother. As in our study the mothers were not matched in terms of their psychological parameters, this could be considered as a limitation for this study.

Ideally, further studies should be performed to gain more insight into the effectiveness of peppermint water. Because the use of a cream or an ointment is easier than a solution, and in order to prescribe a fixed dosage of peppermint water, planning a randomized trial comparing peppermint cream/ointment to no treatment, could be the next step.

## Conclusion

This study suggests that peppermint water is effective in the prevention of nipple crack and the effectiveness is comparable with other remedies. Daily peppermint water use was associated with an increased duration and number of feeds, and less nipple pain compared to the application of expressed breast milk alone. Further studies are needed to assess the usefulness of peppermint water in conjunction with correct breastfeeding techniques.

## Abbreviations

EBM: Expressed breast milk

CS: Cesarean section

RCT: Randomized controlled trial

## Competing interests

The author(s) declare that they have no competing interests.

## Authors' contributions

All authors contributed to the design of the trial, SMM, reviewed the literature, conducted the trial, wrote the first draft of the paper and revised the final draft. RMR and DA, prepared the compound and revised the final draft. ME, GA and KMMH participated in the sequence alignment and drafted the manuscript. SK participated in the design of the study and performed the statistical analysis. TZ participated in education of participants and coordination. All authors read and approved the final manuscript.

## Supplementary Material

Additional File 1 Preparation of the peppermint water. The method for extraction of essential oil, GC-MS analysis and identification of compounds for preparation of peppermint water is discussed. Click here for file
