# Supplementary material for: Effect of peppermint water on prevention of nipple cracks in lactating primiparous women: a randomized controlled trial
Source: Int Breastfeed J. 2007 Apr 19;2:7. doi: 10.1186/1746-4358-2-7 (PMC1865372; doi:10.1186/1746-4358-2-7)
Supplement: Additional File 1 — Preparation of the peppermint water. The method for extraction of essential oil, GC-MS analysis and identification of compounds for preparation of peppermint water is discussed. [file 1746-4358-2-7-S1.doc]

**Additional file**

### Preparation of the peppermint water

**Plant materials**

The aerial parts of *Mentha piperita* were purchased from Hakim Momen Tabrizi Company, and the identity was confirmed by morphological characterization in comparison with the herbarium specimen retained in the School of Pharmacy, Tabriz University of Medical Sciences. Also, a voucher specimen has been generated in that herbarium.

**Extraction of essential oil**

The volatile fraction was obtained by steam distillation for 3 h according to the method recommended in British Pharmacopoeia [1]. The oil was dried over anhydrous sodium sulphate and stored in refrigerator (4ºC). The percentage yield of the essential oil of *M. piperita* was 0.68%.

**GC-MS Analysis**

The essential oil was analyzed by GC-MS using a Shimadzu GCMS-QP5050A gas chromatograph mass spectrometer, DB – 5 capillary columns. Operating conditions were as follows: carrier gas, helium with a flow rate of 0.9 ml/min; column temperature, 3 min in 60 ºC, 60 – 230 ºC at 4 ºC / min and finally 22 min in 230 ºC; injector temperature, 220 ºC; detector temperature, 250 ºC; volume injected, 1 ml of the oil in chloroform (0.1%); split ratio, 1: 45. The MS operating parameters were as follows: ionization potential, 70 eV; ion source temperature, 200 ºC; quadrapole 100 ºC, solvent delay 3.0 min, mass range 25.200 amu, Em voltage 3000 volts [2].

**Identification of compounds**

The identification of the GC peaks corresponding to the components of the essential oil was based on direct comparison of the retention times and mass spectral data with those for standard compounds, computer matching with the NIST NBS54K library, and by comparison of the fragmentation patterns of the mass spectra with those reported in the literature [3].

**Preparation of peppermint water**

The essential oil of peppermint was added to 1 liter of distillated water gradually while the distillated water saturated with it.

**References**

1- Adams RP: *Identification of Essential Oil Components by Chromatography / Mass Spectroscopy.* Allured Publishing Co: Carol Stream; 1995, 18-43, 57-332.

2- Sandra P, Bicchi C, eds: *Capillary Gas Chromatography in Essential Oil Analysis*, Huethig Buch Verlag : Heidelberg; 1987: 259, 274, 287, 328.

3- Swigar AA, Silverstein RM, eds: *Monoterpenes. Infrared, Mass, Proton- NMR, Carbon-NMR Spectra and Kovats Indices***.** Aldrich Chemical Company Inc: Wisconsin; 1981: 3:121.
